# Protic Ionic Liquid Cation Alkyl Chain Length Effect on Lysozyme Structure

**DOI:** 10.3390/molecules27030984

**Published:** 2022-02-01

**Authors:** Qi Han, Hayden C. Broomhall, Nathalia Vieira Veríssimo, Timothy M. Ryan, Calum J. Drummond, Jorge F. B. Pereira, Tamar L. Greaves

**Affiliations:** 1 School of Science, STEM College, RMIT University, 124 La Trobe Street, Melbourne, VIC 3000, Australia or cnhanqi@gmail.com (Q.H.); s3486073@student.rmit.edu.au (H.C.B.); calum.drummond@rmit.edu.au (C.J.D.); 2 School of Pharmaceutical Sciences, São Paulo University (USP), Av. Prof. Lineu Prestes, no. 580, Cidade de Universitária, São Paulo 05508-000, Brazil; nathaliavds@gmail.com; 3 Australian Synchrotron, Australian Nuclear Science and Technology Organisation, 800 Blackburn Road, Clayton, VIC 3168, Australia; timoryan@ansto.gov.au; 4 Univ Coimbra, CIEPQPF, Department of Chemical Engineering, Rua Sílvio Lima, Pólo II-Pinhal de Marrocos, 3030-790 Coimbra, Portugal; jfbpereira@eq.uc.pt

**Keywords:** ionic liquids, protein, lysozyme, alkylammonium nitrate, alkyl chain length, small-angle X-ray scattering (SAXS)

## Abstract

Solvents that stabilize protein structures can improve and expand their biochemical applications, particularly with the growing interest in biocatalytic-based processes. Aiming to select novel solvents for protein stabilization, we explored the effect of alkylammonium nitrate protic ionic liquids (PILs)-water mixtures with increasing cation alkyl chain length on lysozyme conformational stability. Four PILs were studied, that is, ethylammonium nitrate (EAN), butylammonium nitrate (BAN), hexylammonium nitrate (HAN), and octylammonium nitrate (OAN). The surface tension, viscosity, and density of PIL-water mixtures at low to high concentrations were firstly determined, which showed that an increasing cation alkyl chain length caused a decrease in the surface tension and density as well as an increase in viscosity for all PIL solutions. Small-angle X-ray scattering (SAXS) was used to investigate the liquid nanostructure of the PIL solutions, as well as the overall size, conformational flexibility and changes to lysozyme structure. The concentrated PILs with longer alkyl chain lengths, i.e., over 10 mol% butyl-, 5 mol% hexyl- and 1 mol% octylammonium cations, possessed liquid nanostructures. This detrimentally interfered with solvent subtraction, and the more structured PIL solutions prevented quantitative SAXS analysis of lysozyme structure. The radius of gyration (*R_g_*) of lysozyme in the less structured aqueous PIL solutions showed little change with up to 10 mol% of PIL. Kratky plots, SREFLEX models, and FTIR data showed that the protein conformation was maintained at a low PIL concentration of 1 mol% and lower when compared with the buffer solution. However, 50 mol% EAN and 5 mol% HAN significantly increased the *R_g_* of lysozyme, indicating unfolding and aggregation of lysozyme. The hydrophobic interaction and liquid nanostructure resulting from the increased cation alkyl chain length in HAN likely becomes critical. The impact of HAN and OAN, particularly at high concentrations, on lysozyme structure was further revealed by FTIR. This work highlights the negative effect of a long alkyl chain length and high concentration of PILs on lysozyme structural stability.

## 1. Introduction

The in vitro structural stability of proteins is crucially important for a range of applications in the biocatalysis, pharmaceutical, and biomedical areas [1]. However, proteins, especially those used as biocatalysts (i.e., enzymes), often experience various stresses that restrict industrial applications, such as changes in temperature, pH and solvents and the presence of denaturants (e.g., salts, urea, and detergents) [2]. However, there is a need to maintain protein stability in the presence of reduced water, or increased viscosity, for certain biocatalysis reactions. The solvent is one of the most important external factors. A detrimental solvent effect may lead to irreversible protein unfolding, inactivation of enzymes, and/or the formation of aggregates [3]. Therefore, understanding the effect of solvents on protein conformational stability is essential.

Aqueous solutions are the natural solvent for proteins, with other chemical agents often involved for protein stabilization outside their native environment, such as salts, organic solvents, sugars, polyols, and polymers [1]. These additives are often used to improve protein solubility and stability, and in certain cases even to enhance their biological activities [4]. Nonetheless, for many applications, proteins suffer from insufficient protein stability and solubility, and can be affected by the volatility of aqueous solutions [5]. Alternatively, one class of solvents with potential for use with proteins is ionic liquids (ILs). ILs are salts in a molten state that are comprised of ions and exist in a strong pair-wise electrostatic environment [6,7]. ILs have tailorable solvent properties through modification of the cation and anion structures [8,9,10]. Protic ILs (PILs) are a subclass of ILs that are readily tailorable as they are formed simply through proton transfer from a Brønsted acid to a Brønsted base [9,11,12].

There have been a number of prior studies on the effect of ILs on proteins [13,14,15,16]. Notably, IL-water mixtures, from low to high concentrations (i.e., diluted to hydrated), display a range of desirable solvent properties for proteins [16,17,18,19,20]. These aqueous IL solutions were shown to be highly beneficial solvents for stabilizing proteins [21,22], suppressing protein aggregation [23,24], controlling aggregation and crystallization [25,26,27] and improving protein stabilization and biocatalysis [15,19]. As the pH of IL-water mixtures is a critical factor, a few pH-controllable IL systems were utilized, such as IL “buffers” [28], self-buffering ILs [29,30], non-stoichiometric IL-water mixtures [31] and self-buffering IL-water mixtures [23,32,33], which have been previously used with IgY, bovine serum albumin, lysozyme, lipase, and green fluorescent protein.

Moreover, the cation, anion, and cation-anion combinations can have a strong influence on the lysozyme structural stability. For example, EAN and ethanolammonium formate (EtAF) stabilized lysozyme, whereas ethylammonium formate (EAF) and ethanolammonium nitrate (EtAN) were poor solvents, i.e., they have low stabilization ability [31,34]. Studies to date have, however, established that it is difficult to select the ions to achieve particular outcomes due to specific ion effects, local water structure and hydration of proteins, and the complexity of the interactions between the cations, anions, and water [17,18]. 

Previous studies have investigated the effect of increasing the cation chain length of ILs such as 1-alkyl-3-methylimidazolium chloride on protein stability, as changing the alkyl chain length of the cation (as well as of the anion for other IL families) can directly affect the hydrophobicity of the solvent, and hence the hydrophobic interaction with proteins [35,36,37,38]. It was reported that increasing the alkyl chain of 25 *v*/*v*% alkyl-imidazolium chloride up to hexyl decreased the activity and stability of horseradish peroxidase [35]. Venkatesu and co-workers showed that longer alkyl chain lengths in 1-alkyl-3-methylimidazolium ILs led to a major reduction in enzymic activity of stem bromelain owing to hydrophobic interaction [36].

We have previously employed small-angle X-ray scattering (SAXS) and demonstrated that PILs with longer alkyl chain lengths for both cations and anions form liquid nanostructures in the neat form and in the presence of water [39,40,41,42], while the protein size, shape and conformational changes in ILs can be determined [23,27,31]. Despite advances made, there is still a lack of systematic and consistent investigations of the effect of PIL alkyl chain lengths on proteins, and it remains unclear whether increased alkyl chain lengths and associated PIL nanostructures will influence protein behaviour in solutions.

**Figure 1:**
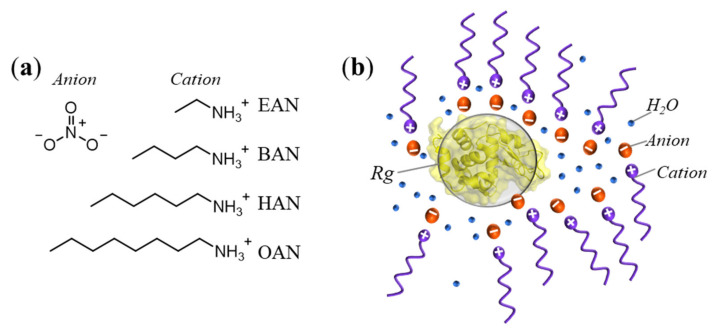
(**a**) Chemical structures of the four chosen PILs for this study, and (**b**) the schematic of lysozyme in the presence of nanostructured HAN as an example. The PILs include ethylammonium nitrate (EAN), butylammonium nitrate (BAN), hexylammonium nitrate (HAN), and octylammonium nitrate (OAN). The correlation distance *d* of HAN and radius of gyration *R_g_* lysozyme were obtained from SAXS experiments, while *d* refers to the average repeat distance of the head groups of PILs separated by their alkyl chains.

In this study, we employ PILs with different cation alkyl chain lengths including ethyl-, butyl, hexyl- and octylammonium nitrate (EAN, BAN, HAN and OAN, respectively) (cf. chemical structures provided in Figure 1a). PIL and alcohol-water mixtures were prepared with low to high concentrations. The density, surface tension, and viscosity were obtained for each PIL-water mixture. Hen egg white lysozyme was used in this work since it is a well-folded globular model protein in water and considered as a near-spherical object [43]. Using SAXS, we simultaneously investigated the liquid nanostructure of the PILs and the effect of these PILs on the size and conformational changes of the model protein lysozyme (cf. schematic representation in Figure 1b). Specifically, the liquid nanostructure, when present, was characterised by a correlation distance *d*, which represents the average repeat distance of the head groups of PILs separated by their alkyl chains, while the radius of gyration *R_g_* was used to evaluate the size of the protein with PILs present. We employed normal-mode analysis to identify the possible conformational changes in the structure of lysozyme in different PIL solutions. Additionally, the secondary structural changes of lysozyme were obtained using FTIR in the PIL solutions.

## 2. Methods

### 2.1. Materials

Ethylamine (70% in methanol), n-butylamine (99.5%), hexylamine (99%), octylamine (99%), nitric acid (70%), ethanol (99.5%), 1-hexanol (99%), and lysozyme from chicken egg white powder (E.C. 3.2.1.17; product code L6878) were obtained commercially from Sigma Aldrich (Burlington, MA, USA). All were used as received.

### 2.2. Sample Preparation

The PILs of ethyl-, butyl-, hexyl- and octylammonium nitrate were all synthesised through a neutralisation reaction of the precursor Brønsted acid/base pair using stoichiometric ratios of the acid (nitric acid) to the base (alkylamine) [40,41,44]. To avoid any issue associated with the highly exothermic nature of this reaction, a drop-wise addition of acid into the base at less than 5 °C was employed. The purity of the PILs is governed by the purity of the starting materials and stoichiometric addition, with water as the main impurity. Excess water was removed from each PIL using a rotatory evaporator (Heidolph Hei-VAP Core, Schwabach, Germany) at 40 °C, followed by a LabconcoFreeZone 4.5 Litre Freeze Dry System (Labconco, Kansas City, MO, USA) until less than 1 wt% water (>99% purity) was present, as measured using a coulometric Karl Fischer titrator (Mettler Toledo DL390, Columbus, OH, USA). The obtained EAN and BAN samples were transparent liquids while HAN and OAN were transparent and gel-like, as described previously [41].

The solutions were prepared by diluting each PIL with MilliQ water (MilliporeSigma, Burlington, MA, USA) to obtain 0.1, 0.5, 1, 5, 10, 20 and 50 mol% PIL (Table S1, Supplementary Materials). The aqueous PIL solutions with concentrations below 20 mol% were pH adjusted to 8 ± 0.2 using a concentrated tris buffer solution as previously described [27,33]. Hydrogen ion activity (pH) measurement in non-aqueous solvents, such as neat ILs, is not a simple matter of employing a pH electrode-meter, which has been calibrated with aqueous buffer systems [45]. In addition, at high concentration, i.e., over 20 mol%, most hydrophilic ILs have strong inter and intra-molecular interactions with water to form ionic pairs or clusters, and there is no free water present in the solution [17,20,46]. Lysozyme solutions were prepared through dilution of a lysozyme stock solution (100 mg/mL lysozyme in Tris buffer, 100 mM, pH 8) to reach concentrations of 5 mg/mL for SAXS and 20 mg/mL for FTIR analyses. After the addition of the protein solution, the solution was mixed by repeated pipetting, followed by vortexing to ensure the protein was homogeneously suspended in the solvents, as previously described [24].

### 2.3. Analytical Characterisation

The surface tension, viscosity, and density were measured for all PIL aqueous solutions at 20 ± 2 °C. The surface tension was measured using a Kibron Delta-8 surface tension plate reader (Kibron Inc., Helsinki, Finland), with samples loaded into 96 well plates. Density was measured using an Anton Paar DMA 4500 M densiometer (Graz, Austria), with 2 mL of each solution inserted into the instrument. Solution viscosity was measured using an A&D SV-1 A viscometer (Tokyo, Japan) on 2 mL of each solution at 20 ± 2 °C.

FTIR spectra were acquired using a Perkin-Elmer Frontier FTIR Spectrometer (Waltham, MA, USA) with an ATR attachment at room temperature. All samples were equilibrated with lysozyme present (20 mg/mL) for 1 h before acquisition. Each spectrum consisted of 64 scans with a resolution of 4 cm^−1^. Careful background subtraction with each solvent was performed to minimize solvent contributions, while the spectra were smoothed (Savitzky-Golay, polynomial degree 2, points of window 50) and normalized to the same intensity for ease of spectral comparison.

SAXS experiments were completed at the Australian synchrotron SAXS/WAXS beamline, using the automated well plate system (autoloader) [47]. The SAXS data were recorded on a Pilatus2-1M silicon photon-counting detector (Baden, Switzerland) with an incident beam of wavelength λ = 1.033 Å (12.0 keV) and a typical flux of around 10^13^ photons/s. The *q* range for the setup was ~0.01 to 0.55 Å^−1^ with a sample-detector distance of 2.4 m. Due to the instrumental background noise close to the beamstop, the first five data points were not used for any analysis. Portions of each sample with and without lysozyme present (5 mg/mL, 100 μL) were loaded into 96-well plates six hours before the experiments, as reported in our previous work [24]. During SAXS experiments, the autoloader automatically loaded each sample into a double-ended 1 mm capillary. Twenty successive frames of 1 s exposure were collected for each sample and buffer, while it was under flow in order to minimize radiation damage. Washing routines were conducted between samples, and the same capillary was used for all samples to improve the solvent subtraction. Scatterbrain 2.82 was used for averaging multiple SAXS patterns, and for solvent subtraction [48]. The SAXS patterns for IL-water solvent samples were obtained first. A broad peak in the *q* range of 0.1 and 0.55 Å^−1^ for IL-water samples indicates the presence of a liquid nanostructure through the segregation of polar and non-polar moieties. Where this peak was present, a correlation length *d* was approximated from the peak position using Bragg’s law, *d* = 2π/*q*_max_. For SAXS patterns of the protein in ILs, accurate solvent subtraction was performed by matching the scattering intensities of the solvent and protein samples by applying a multiplication factor of ~0.99 to the solvent. However, no accurate solvent subtraction was possible for proteins in ILs with a broad liquid nanostructures peak (Figure S1, Supplementary Materials) due to small variations in the IL peak shape upon the addition of proteins. All the patterns are presented with a vertical offset for comparison in the figures. From the protein SAXS patterns, the radius of gyration *R_g_* was calculated using the Guinier approximation (where *qR_g_* < 1.3 Å), obtained through the ATSAS package (EMBL, Hamburg Germany), where *R_g_* is a measurement of the root-mean-square distance of each atom from the center of the protein molecule. Kratky plots were plotted of *q*^2^*I* against *q*, where *q* is the scattering vector, and *I* is the scattering intensity. CRYSOL module in ATSAS was used to compare experimental SAXS scattering with the model scattering based on the lysozyme crystal structure (PDB ID 7jmu with lysozyme crystallized in 1 mol% EAN [27]). SREFLEX(EMBL, Hamburg Germany) was then used for refinement and normal mode analysis from the initial structure (7jmu) [49], and the best refined models in different ILs were selected based on the χ^2^ value. An open-source PyMOL v. 1.8.4.0 (PyMOL Molecular Graphics System, Version 1.8.4.0, Schrödinger, New York, NY, USA) was used to visually analyze and generate figures.

## 3. Results and Discussion

### 3.1. Physicochemical Properties of PIL-Water Mixture

**Figure 2:**
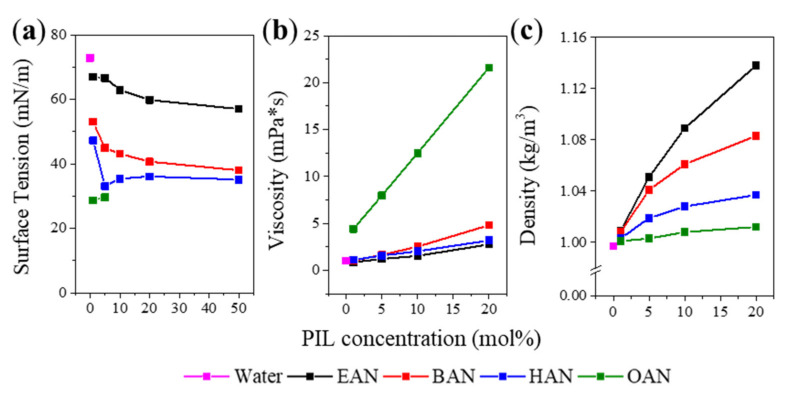
Physicochemical properties of PIL solution as a function of PIL concentration (0 mol% corresponds to water) measured at 20 °C for (**a**) surface tension, (**b**) viscosity and (**c**) density.

The surface tension, density, and viscosity for each PIL-water mixture were measured, and the effect of increasing cation alkyl chain length and the concentration of PILs on the properties is shown in Figure 2. These three properties were characterised as the surface tension and viscosity have been shown to affect the stabilization of globular proteins [50,51], and because density is likely to be necessary for any future computational studies on these solutions. The high viscosity and low surface tension of the OAN samples limited the viable surface tension measurements that could be made on the 1 and 5 mol% OAN solutions. 

Figure 2a shows that the air-liquid surface tension of all four PIL-water mixtures generally decreased with increasing PIL concentration, with the surface tension of water (72 mN/m) included for comparison. The presence of 1 mol% PIL led to a significant decrease in the surface tension, relative to water, and was roughly proportional to the increased alkyl chain length present on the cation, showing that these cations are surface active. Increasing the IL concentration from 1 mol% to 50 mol% led to the PIL solutions becoming less water-like, decreasing by ~10 mN/m for EAN, BAN and HAN. Notably, there was a decrease and then a plateau at 5 mol% for BAN and HAN, which is likely related to the formation of nanostructure assemblies. The surface tension of OAN above 5 mol% was too low to be measured using the instrument, and little difference was observed between 1 and 5 mol% OAN. Thus, increasing the alkyl chain length had a greater impact on the solution surface tension than the IL concentration, due to their amphiphilic properties [42].

Figure 2b shows that increasing the PIL concentration increased the solution viscosity. It is noticeable that the viscosity of OAN solutions increased significantly more relative to the three shorter chained PILs. This increase is attributed to the longer alkyl chains leading to increased cation entanglement in the solvent [52], and to a greater liquid nanostructure through enhanced segregation of polar and non-polar regions. We have previously reported that neat OAN forms a liquid crystalline lamellar phase, and it is anticipated that this structure will be retained on addition of water until some threshold is met [44], with increased van der Waals interaction occurring between the alkyl chains relative to the shorter chained PILs. Although some small differences in viscosity can be found for EAN, BAN and HAN, particularly at 20 mol%, these three PIL solutions are far less viscous than the OAN solutions, which have a viscosity ranging from 2 to 5-fold higher compared with the other solutions (as a function of the PIL concentration).

Figure 2c shows the solution density, which is an indicator of how well packed the different PILs are within the solution. The density increased with the increasing PIL concentration, though not linearly. At each concentration, the density followed the series of EAN > BAN > HAN > OAN. EAN solutions had the highest densities among these PILs, which was attributed to the smaller ion sizes. Smaller ions can be more tightly packed together, as well as the more hydrophilic cation due to the lower alkyl proportion, which is likely to lead to stronger solvation of the cation with water. The concentrated solutions (20 mol%) showed a considerable difference between EAN with a density of 1.13 and OAN with a density of 1.01 kg/m^3^. This alkyl chain-dependent effect has also been witnessed using aprotic IL anions. showing that both the cation and anion can impact the solution packing efficiency [53].

### 3.2. Liquid Nanostructure

Many neat ILs form a short to medium range order liquid nanostructure which is formed through segregation of the polar and non-polar parts of the anions and/or cations [42,54,55], which is analogous to amphiphilic self-assembly structures in a liquid and widespread in the crystal structure of conventional amphiphiles. This liquid nanostructure is often heterogeneous in nature because ILs are composed of two components, anions and cations, or, alternatively, charged and uncharged moieties. In addition, this nanostructure can be retained upon the addition of water, and affects how ILs will interact with solutes [56,57]. 

**Figure 3:**
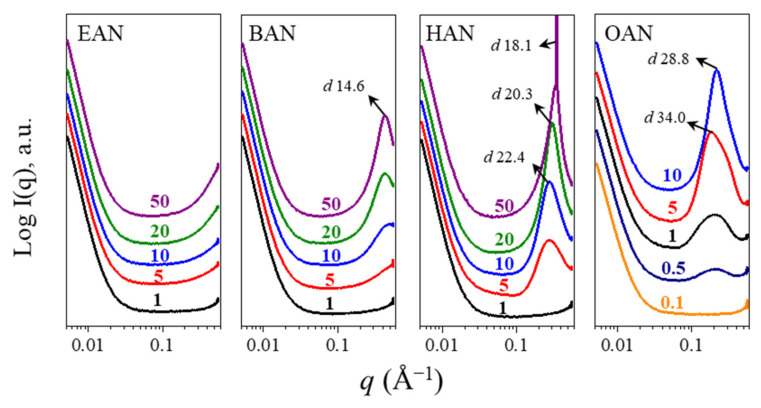
The SAXS profiles of EAN, BAN, HAN, and OAN-water mixtures with correlation distances (*d*), indicated by the arrows. The color numbers on the plots refer to the PIL concentration in mol%.

The liquid nanostructure for each PIL solution was characterized using SAXS in the *q* range above 0.01 Å^−1^ (Figure 3). A broad peak was observed for certain BAN, HAN and OAN solutions, indicating that a liquid nanostructure was present. These nanostructures were observed in BAN, HAN and OAN solutions at concentrations of over 10, 5, and 0.5 mol%, respectively, which shows that the nanostructure is robust to dilution, particularly for the longer chained OAN. These broad peaks were observed in the *q* range of 0.01–0.5 Å^−1^, and can affect the interpretation of lysozyme scattering patterns, as discussed in the following section. For solutions of 5 mol% or more EAN and 5 mol% BAN there was an increase in the scattering for *q* greater than 0.1 Å^−1^, indicating some liquid nanostructure may be present, but these patterns could still be analysed.

An approximate correlation distance (*d*) was obtained from Bragg’s law based on the peak position (*q_peak_*) of this broad peak using *d* = 2π/*q_peak_* and, when present, is included in Figure 3. This correlation distance is attributed to the repeat distance from the charged headgroup on one ion to the charged headgroup on the second ion, separated by their cation alkyl chains [41]. However, due to the lack of long-range ordered nanostructures, no clear mesophase (usually displays as a sharp peak in SAXS profile) can be identified. As expected, *d* increases with the increasing alkyl chain length on the cation. The *d* value for BAN was consistent with that of neat BAN of 14.02 Å [42], and similar to previous findings for PILs with an alkyl chain of 5 carbons or less (*d* of pentylammonium nitrate was 15.94 Å), had little change with the changing water content, though the intensity decreased with the increasing water content [40].

For HAN and OAN there was a decrease in *d* with the increasing water content, which suggests there is a different interaction with water for the longer chained PILs, compared with the shorter ones. Additionally, a sharp peak was present in 50 mol% HAN, which is likely due to the formation of a lamellar liquid crystal phase, with significantly more structure and longer-range order than the other PIL solutions. It is expected that a similar lamellar phase would be present for higher concentrations of the OAN aqueous solutions, particularly since 20 and 50 mol% OAN could not be used due to gel-like high viscosities of the solutions. Previously, we have reported that both neat HAN and OAN form a thermotropic lamellar liquid crystal phase, whereas alkylammonium nitrates with 5 carbons or less in their chains did not [44]. The liquid nanostructure *d* for neat HAN at 60 °C was reported to be 18.5 Å^−1^ [44], and the *d* values for HAN and OAN at 25 °C were reported to be 17.6 and 21.2 Å^−1^, respectively [44]. These previous values are comparable to the PILs aqueous solutions of this work, and support the finding that *d* increases with increasing water proportion for alkylammonium nitrate PILs with alkyl chains longer than 5 carbons.

### 3.3. Conformational Changes of Lysozyme 

**Figure 4:**
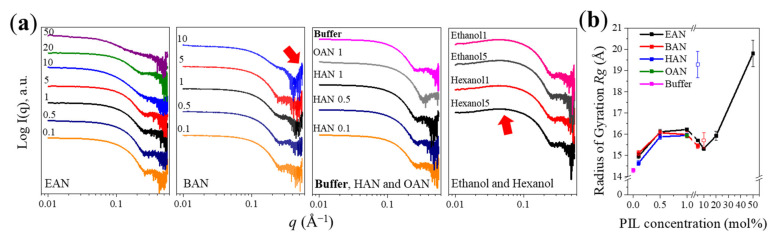
SAXS profiles of lysozyme in different solvents (i.e., EAN, BAN, HAN, OAN, buffer, ethanol and hexanol-water mixtures) (**a**) and the *R_g_* values as a function of PIL concentration (**b**). The numbers on the plots refer to the PIL and alcohol concentration in mol%. The arrow in BAN 10 mol% solution illustrates the contribution of PIL nanostructure, while the arrow in ethanol and hexanol solutions refers to the downturn at low *q* owing to the repulsive inter-particle interactions. The *R_g_* of lysozyme in 10 mol% BAN and 5 mol% HAN solutions (open square) is an estimate, since the SAXS patterns were affected by subtraction issues due to PIL nanostructure (Figure S2, Supplementary Materials). The Guinier plots used for calculating *R_g_* are provided in Figure S3 (Supplementary Materials), and the data analysis details are presented in Table S2 (Supplementary Materials).

The SAXS patterns of lysozyme (solvent subtracted) in the PIL solutions are provided in Figure 4, along with those in ethanol and hexanol solutions for comparison purposes. However, due to the presence of the broad peak of PIL nanostructure within the *q* region (cf. Figure 3), accurate solvent subtraction was not achieved in solutions containing 10–50 mol% BAN, 5–50 mol% HAN, or 5–10 mol% OAN. This is because there were small changes to the shape of the broad peak with lysozyme present, attributed to the nanostructure of the PIL becoming modified by the presence of lysozyme, causing a broader range of correlation distances. The effect on the broad peak in the SAXS patterns is shown in Figures S1 and S2 of the Supplementary Materials. An example of the resulting issue with subtraction is included in Figure 4a for lysozyme in BAN 10 mol%, where it is apparent that for the *q* region above 0.3 Å^−1^ there is a decrease in intensity followed by an upturn in intensity (indicated by the red arrow in Figure 4a). This feature is an artefact from solvent subtraction due to the liquid nanostructure of the PIL, as this region should have effectively no scattering, and no features, as shown for the subtractions for lysozyme in the buffer solution in Figure 4a. This effect becomes more pronounced with increasing nanostructure, as shown in Figure S2 (Supplementary Materials) for lysozyme in 10, 20 and 50 mol% BAN solutions. Perhaps, most importantly, this leads to an increased likelihood of poor subtraction over other *q* regions, since part of the criteria for good solvent subtraction for SAXS patterns of proteins in aqueous solutions is achieved by matching the scattering intensity of the sample and water over this high *q* region. Consequently, solvent subtraction without artefacts was only achievable for lysozyme in EAN at 0.1–50 mol%, BAN at 0.1–5 mol%, HAN at 0.1–1 mol% and OAN at 1 mol%. Compared with the lysozyme SAXS profile in buffer, lysozyme in the PIL solutions shown in Figure 4a, presented similar features with a flat region at a low *q* indicative of monomeric protein, followed by a decrease in scattering intensity at a higher *q*. 

To explore whether the effect of the alkyl chain length on the PILs was due to their amphiphilic properties, ethanol and hexanol aqueous solutions with the same alkyl chain length as EAN and HAN were used for comparison. However, a decrease in intensity at low angles was observed for all the alcohol solutions at 1 and 5 mol%. This stems from the repulsive inter-particle interactions and can lead to an underestimation of *R_g_* [58], while the corresponding Guinier plot (the “frowning” Guinier) is shown in Figure S3 (Supplementary Materials). This demonstrates that the nature of the solvent, i.e., molecular or ionic solvent, is more critical than the alkyl chain length. Additionally, such “frowning” Guinier behavior was not observed in IL-water mixtures previously [27].

As shown in Figure 4, lysozyme maintained a monomeric state in most of the aquous PIL solutions, and hence, the radius of gyration *R_g_* could be obtained to identify changes in the size of lysozyme in the PIL solutions compared with the buffer solution. Figure 4b shows the *R_g_* changes as a function of PIL concentration, and Figure S3 (Supplementary Materials) shows the corresponding Guinier plots which were used for calculating *R_g_*. It should be noted that the *R_g_* value for lysozyme in 50 mol% EAN only provides an estimated *R_g_*, i.e., pseudo-*R_g_*, which was described previously and is used for aggregated proteins [24]. Tentative *R_g_* values were obtained for lysozyme in 10 mol% BAN and 5 mol% HAN solutions, where the solvent nanostructure affected the subtraction, but a reasonable SAXS pattern was obtained (included in Figure 4b using open symbols). The *R_g_* of lysozyme within a buffer solution (i.e., 0 mol% PIL) was 14.3 Å, and it increased with the addition of the PILs. In particular, *R_g_* slightly increased to ~15 Å with 0.1 mol% EAN, BAN or HAN present, and then increased to ~16 Å at 0.5 and 1 mol% PILs. Interestingly, a slight decrease of *R_g_* from ~16 to ~15 Å was observed on going from 5 to 10 mol%, though this is only based on data in EAN, and tentative data in BAN, since the other PILs could not be used due to nanostructure issues outlined above. This increase in *R_g_* at 1 mol% is possibly owing to the strong interaction between lysozyme and the nitrate ions leading to an increased hydration shell and expansion of the loop regions [27].

With 50 mol% EAN present, the Guinier region showed a significant increase in intensity at a low *q*^2^ (Figure S3, Supplementary Materials), which is indicative of aggregates being present. An approximate *R_g_* of lysozyme was obtained from the linear region, which was ~20 Å, also consistent with the existence of aggregates. This aggregation is possibly caused by the high ionic strength and strong interactions of nitrate ions with the surface of lysozyme, suggesting that the electrostatic interaction of nitrate ions with the surface of lysozyme was also dominating. The *R_g_* was directly increased to ~19 Å in 5 mol% HAN solution. This effect confirms that the hydrophobic interaction and liquid nanostructure resulting from the increase of cation alkyl chain for HAN likely becomes critical, leading to lysozyme unfolding and aggregation with the solution at 5 mol% HAN. This negative effect on lysozyme structure was not observed in 5 mol% EAN and BAN solutions. 

**Figure 5:**
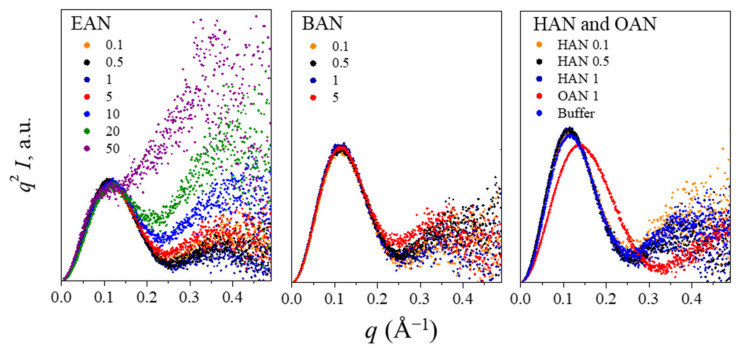
Normalized Kratky plots of lysozyme in PIL aqueous solutions (i.e., EAN, BAN, HAN and OAN), along with those in a buffer solution for comparison.

Kratky plots were obtained to show the flexibility of lysozyme with the PILs present and are shown in Figure 5. The bell-shaped curves were observed in all the PIL solutions with a concentration lower than 10 mol% (where the data could be obtained through subtraction), corresponding to the protein being folded and retaining the globular shape of lysozyme. The deviation from the bell-shaped curve for *q* > 0.1 Å^−1^ can be seen with increasing EAN concentration. The large deviation from the bell-shape for 20 and 50 mol% EAN suggests at least partial unfolding of the lysozyme structure and is consistent with the changes to the shape of the scattering curve shown in Figure 4a for these samples. The Kratky plots for lysozyme in the BAN solutions showed a similar change as EAN solutions at same concentrations, while a small change in the bell-shape was observed with the solution of BAN at 5 mol%. Regarding HAN and OAN, little change was seen for 0.1–1 mol% HAN, whereas a noticeable shift was found for OAN 1 mol%. This shift indicates that long alkyl chains on the PIL cation may negatively affect lysozyme flexibility, with the octyl chain having a significant negative impact compared with the shorter chained cations.

**Figure 6:**
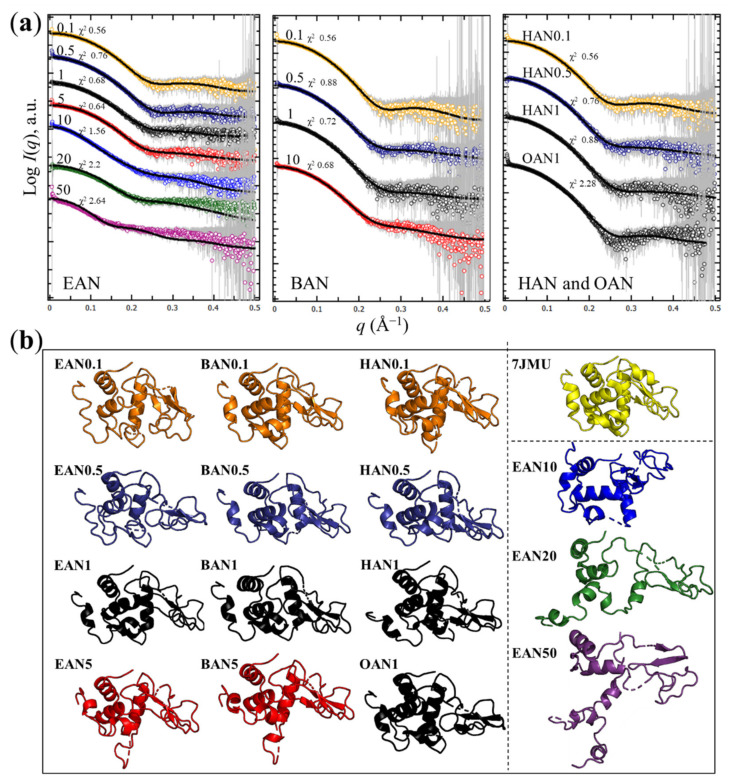
(**a**) SREFLEX fits of SAXS data of lysozyme in PIL-water mixtures (EAN, BAN, HAN and OAN with concentration (mol%) provided), where the solid line refers to the fitting after SREFLEX refinement. The refined model was selected based on the χ^2^ values closest to 1.00 which indicates the best fit, and the values are included on the plots. (**b**) The resulting SREFLEX models of lysozyme in PIL-water mixtures at different PIL concentrations (mol%), and the initial crystal structure of lysozyme (yellow, PDB 7jmu).

To gain further insights into the conformational change of the lysozyme in PIL solutions, the high-resolution lysozyme crystallographic structure (PDB: 7jmu, crystallized in EAN 1 mol%) was fitted to the SAXS data followed by refinement to improve the agreement with the SAXS profiles using normal-mode analysis, SREFLEX [49]. The lysozyme structures obtained by SREFLEX can identify possible conformational changes of lysozyme in the presence of different PIL solutions, as shown in Figure 6. The discrepancy χ^2^ values of the initial lysozyme model (yellow structure in Figure 6b) fitted to the SAXS data and the χ^2^ values after the refinement using SREFELX are provided in Figure 6a. The fit quality was reduced for the samples with the largest deviation in structure, namely for lysozyme in 20 and 50 mol% EAN, and in 1 mol% OAN.

It is apparent that, at PIL concentrations from 0.1 to 1 mol% the overall secondary and tertiary structure of the lysozyme was maintained. However, in the presence of a solution with 5 mol% EAN or BAN, a reorientation in the α-helix regions and some disordered regions can be observed. These changes might be leading to the expansion of lysozyme size and the increase in the flexibility of lysozyme which were seen in Figure 4b and Figure 5, respectively. A further increase in the EAN concentration to 10 mol% led to more substantial changes in the α-helix region. The expansion of lysozyme structure was noticeable with 20 and 50 mol% EAN present, indicating that lysozyme has unfolded. Unfortunately, the liquid nanostructure of the BAN, HAN and OAN solutions prevented the SREFLEX analysis from being performed on lysozyme in higher concentrations of these PILs, though it is interesting that solutions with 1 mol% of these PILs have minimal effects.

**Figure 7:**
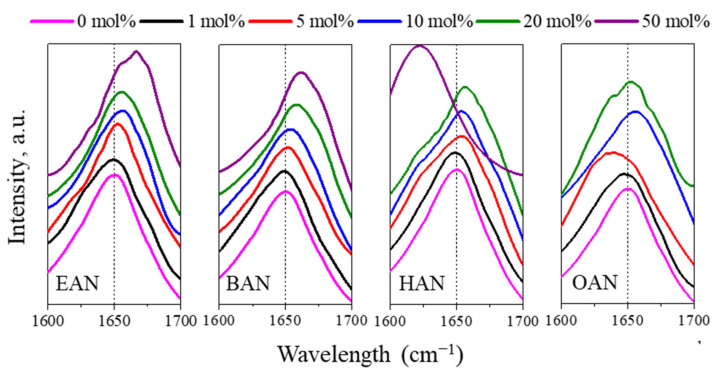
Normalised FTIR spectra of lysozyme in PIL-water mixtures (EAN, BAN, HAN and OAN). The control sample of lysozyme in a buffer solution at a pH value of 8 (0 mol% of PIL) is included for comparison in each series. The dashed line refers to the peak of the amide I band in buffer.

To further investigate the secondary structure changes, FTIR spectroscopy was used to characterise changes to the amide I band (C=O stretching modes, 1600–1700 cm^−1^) of lysozyme in PIL aqueous solutions with concentrations from 1 to 50 mol% of PIL. The FTIR spectra are shown in Figure 7, and for EAN, BAN and HAN solutions the amide I peak at 1650 cm^−1^ was shifted to higher wavenumbers with the increasing PIL concentration. The increase in absorbance around 1660 cm^−1^ is likely due to anti-parallel β-sheet formation [27]. These absorbance shifts as a function of PIL concentrations are consistent with the conformational changes observed by SREFLEX models where lysozyme tended to gradually lose structure with the increasing PIL concentrations for EAN and BAN (cf. Figure 6). Comparing the four PILs, no significant change in the amide I band shift (peak at 1650 cm^−1^) was observed with 1 mol% of PIL solution, while the presence of 10 mol% or more of the PILs led to noticeable secondary structural changes. This is consistent with our previous studies using other PILs such as those containing alkylammonium cations, alkanolammonium cations, secondary ammonium cations, carboxylate anions or mesylate anions [27,34].

The FTIR spectra of lysozyme in 50 mol% HAN was noticeably different, and this can be attributed to the presence of the lamellar liquid crystal phase (evident from the SAXS pattern of the HAN aqueous solution in Figure 3), causing a significant change to the amide I band. As for OAN, a noticeable blueshift was observed even at 5 mol%, indicating that the longer alkyl chain length may lead to the formation of β-turns [59]. This change is opposed to the redshift in the other three PILs, indicating the possible strong hydrophobic interaction, which was not detected by the SAXS data. The higher concentrations of 10 and 20 mol% OAN led to similar peak shifts as the other PILs, but with a much broader peak. This additional feature suggests substantial changes in the secondary structure including the loss of helical structure and β-turn re-arrangements [59].

With 50 mol% of EAN, BAN and HAN present a significant shift was identified. The high viscosity of 50 mol% OAN prevented the lysozyme addition, and it was not measured. The concentrated PILs with longer alkyl chain lengths, i.e., HAN and OAN, seem to have a different influence on the secondary structure of lysozyme than EAN and BAN, which is attributed to hydrophobic interactions in between cation and hydrophobic regions at the surface of lysozyme. These results are consistent with previous reports showing that higher alkyl chain length imidazolium-based ILs have a more denaturing effect on the structure of stem bromelain compared with the lower alkyl chain length ILs, while low concentrations (0.01–0.10 M) of short alkyl chain imidazolium-based ILs maintain the protein structure [36].

## 4. Conclusions

This work investigated the solvent properties of aqueous solutions of four alkylammonium nitrate PILs with cation alkyl chain lengths of 2, 4, 6 and 8 carbons, and their influence on lysozyme conformational stability. This expands on previous studies using imidazolium-based ILs with long or short alkyl chain lengths at low concentrations in water. The results show that the surface tension of EAN, BAN and HAN solutions decreased as a function of PIL concentration, while their viscosity and density increased. However, the properties of OAN-water mixtures were different, with the viscosity increasing significantly with the increasing PIL concentration. SAXS was used to characterise the PIL nanostructure and lysozyme structure in the PIL solutions. SAXS patterns of the PIL solutions confirmed the presence of liquid nanostructures in solutions with at least 10 mol% BAN, 5 mol% HAN and 1 mol% OAN in the aqueous solutions. Such liquid nanostructure is represented by the correlation distance (*d*) with the longer alkyl chain lengths showing higher *d* values. Based on SAXS data, the protein flexibility and conformational changes in the PILs were identified, showing that lysozyme structure was maintained at 0.1, 0.5 and 1 mol% PILs. Similarly, the *R_g_* of lysozyme slightly increased to ~16 Å from 15 Å with the addition of 0.5 mol% or 1 mol% of PILs. This suggests that there is a minimal effect from the cation alkyl chain length on the protein size at low PIL concentrations, which is attributed to the anion having a much greater effect on the protein structure at low PIL concentrations. For PIL concentrations of ≥5 mol%, only a few SAXS patterns could be analysed due to issues with solvent subtraction from the liquid nanostructure. However, it was observed that the *R_g_* of lysozyme remained at ~16 Å with the helical structure of lysozyme altered in 5 or 10 mol% EAN and BAN. The *R_g_* significantly increased to ~20 Å in 50 mol% EAN and 5 mol% HAN with lysozyme structure becoming partially unfolded. FTIR data showed a noticeable change in the secondary structure of the protein with the increasing IL concentration, and that the ILs with longer chains of HAN and OAN appear to change the secondary structure. These results indicate a greater influence from the longer-chain cations of HAN and OAN at higher PIL concentrations owing to the hydrophobic interaction and enhanced liquid nanostructure. This work highlights the impact of cation alkyl chain length on protein structural stability and has significant implications for designing solvents for protein stabilization and biocatalysis.

## Data Availability

Not available.

## Supplementary Materials

Figure S1: Examples of solvent subtraction in SAXS profile for PIL-water mixtures (the solvent sample) with the broad peak due to the liquid nanostructures. Accurate solvent subtraction was not achieved for BAN 20 and 50 mol%, HAN 5–50 mol%, or OAN 5–10 mol%. The arrows indicate the unmatched high *q* region which render subtraction prone to artefacts, Figure S2: SAXS profiles of lysozyme in concentrated BAN and HAN solutions with large contribution of liquid nanostructure of the PIL shown in the grey area. The numbers on the plots refer to the PIL concentration in mol%. The details for estimating *R_g_* are provided in Table S2, Figure S3: Guinier plots of lysozyme in the PIL-water mixtures compared with buffer. The range for calculating R_g_ values are obtained from the ATSAS 3.01 software. The detailed parameters were provided in Table S2. An offset was applied for easier comparison of the samples. The arrow refers to the Guinier plot (the “frowning” Guinier) owing to the repulsive inter-particle interactions in ethanol and hexanol solutions. The numbers on the plots refer to the PIL concentration in mol%, Table S1: Weight percentage (wt%) of PILs corresponding to molar concentrations (mol%) in PIL-water mixtures in this study, Table S2: Details of SAXS parameters of data analysis.
