# Supplementary material for: Protic Ionic Liquid Cation Alkyl Chain Length Effect on Lysozyme Structure
Source: Molecules. 2022 Feb 1;27(3):984. doi: 10.3390/molecules27030984 (PMC8839406; doi:10.3390/molecules27030984)
Supplement: Supplementary file 1 [file molecules-27-00984-s001.zip › molecules-1522452-supplementary.pdf]

molecules MDPI

Supplementary Materials

Protic Ionic Liquid Cation Alkyl Chain Length Effect on Lysozyme Structure

Qi Han [1,†], Hayden C. Broomhall [1,†], Nathalia Vieira Veríssimo [2], Timothy M. Ryan [3], Calum J. Drummond [1], Jorge F. B. Pereira [4] and Tamar L. Greaves [1,*]

[1] School of Science, STEM College, RMIT University, 124 La Trobe Street, Melbourne, VIC 3000, Australia; qi.han@rmit.edu.au; cnhanqi@gmail.com (Q.H.); s3486073@student.rmit.edu.au (H.C.B.); calum.drummond@rmit.edu.au (C.J.D.).
[2] School of Pharmaceutical Sciences, São Paulo University (USP), Av. Prof. Lineu Prestes, no. 580, Cidade de Universitária, 05508-000 São Paulo, Brazil; nathaliavds@gmail.com.
[3] Australian Synchrotron, Australian Nuclear Science and Technology Organisation, 800 Blackburn Road, Clayton, VIC 3168, Australia; timoryan@ansto.gov.au.
[4] Univ Coimbra, CIEPQPF, Department of Chemical Engineering, Rua Sílvio Lima, Pólo II-Pinhal de Marrocos, 3030-790 Coimbra, Portugal; jfbpereira@eq.uc.pt.
* Correspondence: tamar.greaves@rmit.edu.au.
† These authors contributed equally to this work.

Table S1. Weight percentage (wt%) of PILs corresponding to molar concentrations (mol%) in PIL-water mixtures in this study.

PILs	Molecular Weight	PIL Concentration in mol%						
		0.1	0.5	1	5	10	20	50
EAN	108.1	0.6	3	5.7	24	40	60	85.7
BAN	136.1	0.8	3.6	7	28.6	45.7	65.4	88.4
HAN	164.2	1	4.4	8.4	32.5	50.3	69.5	90.1
OAN	192.2	1.1	5.1	10	36	54.3	72.8	91.5

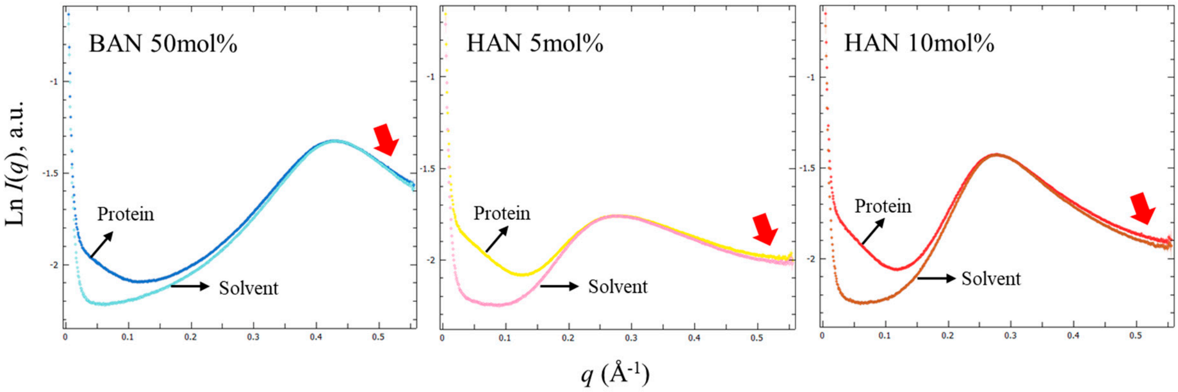

Figure S1. Examples of solvent subtraction in SAXS profile for PIL-water mixtures (the solvent sample) with the broad peak due to the liquid nanostructures. Accurate solvent subtraction was not achieved for BAN 20 and 50 mol%, HAN 5–50 mol%, or OAN 5–10 mol%. The arrows indicate the unmatched high q region which render subtraction prone to artefacts.

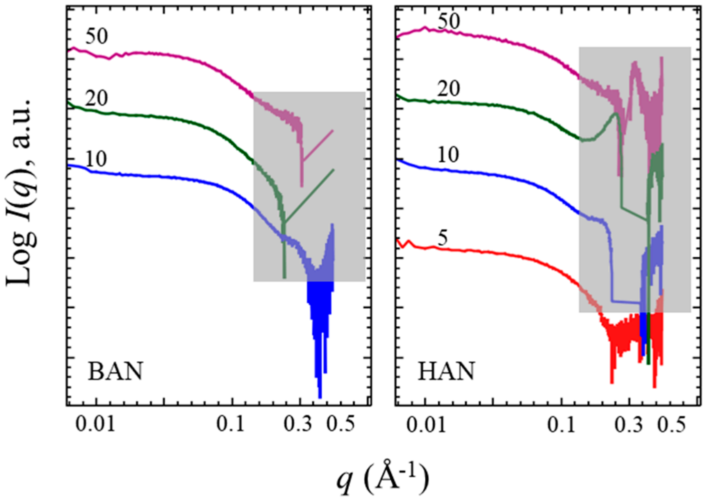

Figure S2. SAXS profiles of lysozyme in concentrated BAN and HAN solutions with large contribution of liquid nanostructure of the PIL shown in the grey area. The numbers on the plots refer to the PIL concentration in mol%. The details for estimating R_g are provided in Table S2.

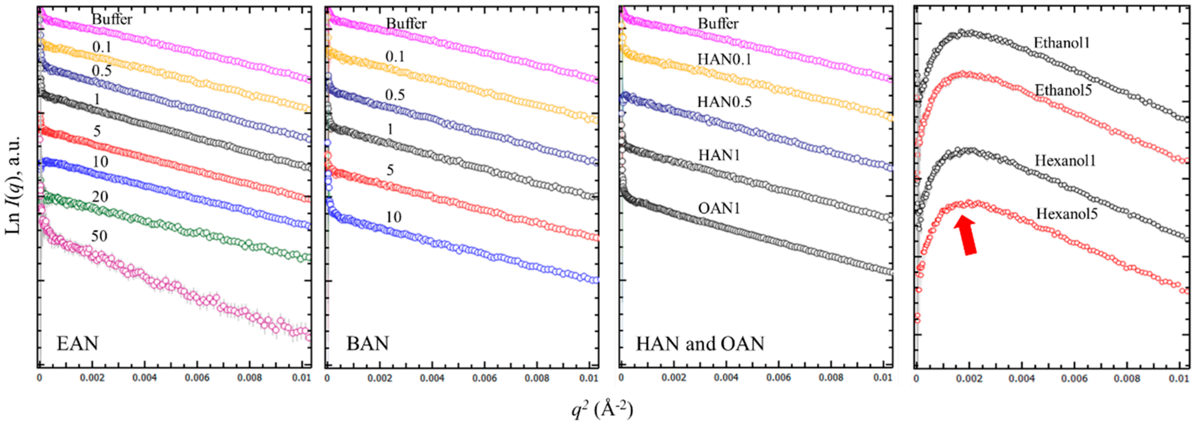

Figure S3. Guinier plots of lysozyme in the PIL-water mixtures compared with buffer. The range for calculating R_g values are obtained from the ATSAS 3.01 software. The detailed parameters were provided in Table S2. An offset was applied for easier comparison of the samples. The arrow refers to the Guinier plot (the "frowning" Guinier) owing to the repulsive inter-particle interactions in ethanol and hexanol solutions. The numbers on the plots refer to the PIL concentration in mol%.

Table S2. Details of SAXS parameters of data analysis.

PIL-Water Mixtures (mol%)	Guinier R_g (Å) [std.err]	qR_g (max)	Data Points for Calculating *Rg*
**pH 8**	14.27 ± 0.09	1.21	0.01
**EAN0.1 mol%**	14.96 ± 0.11	1.29	9–102
**EAN0.5 mol%**	16.11 ± 0.13	1.25	18–91
**EAN1 mol%**	16.22 ± 0.08	1.29	20–118
**EAN5 mol%**	15.71 ± 0.07	1.29	15–122
**EAN10 mol%**	15.32 ± 0.08	1.29	19–125
**EAN20 mol%**	15.22± 0.21	1.3	9–103
**EAN50 mol%**	19.81 ± 0.62	1.29	20–76
**BAN0.1 mol%**	15.11 ± 0.12	1.29	17–101
**BAN0.5 mol%**	16.07 ± 0.12	1.28	15–94
**BAN1 mol%**	15.98 ± 0.14	1.29	11–95
**BAN5 mol%**	15.44 ± 0.11	1.29	14–99
**BAN10 mol%** *	15.71 ± 0.37	0.95	19–70
**BAN20 mol%** *	20.86 ± 0.33	1.26	19–70
**BAN50 mol%** *	20.87 ± 0.50	1.26	19–70
**HAN0.1 mol%**	14.64 ± 0.12	1.3	16–105
**HAN0.5 mol%**	15.88 ± 0.12	1.28	10–95
**HAN1 mol%**	15.94 ± 0.11	1.27	16–94
**HAN5 mol%** *	19.29 ± 0.62	1.17	19–70
**HAN10 mol%** *	21.85 ± 0.26	1.32	19–70
**HAN20 mol%** *	20.62 ± 0.45	1.25	19–70
**HAN50 mol%** *	23.91 ± 0.58	1.45	19–70
**OAN1 mol%**	15.93 ± 0.10	1.3	64–121

* The R_g of lysozyme in concentrated BAN and HAN solutions was estimated by the SAXS pattern (Figure S2) where specified data points for calculating R_g was employed.
